# Highlights of the 3rd ISCB Africa Student Council Symposium 2019 in Ghana

**DOI:** 10.12688/f1000research.24101.1

**Published:** 2020-05-27

**Authors:** Wisdom A. Akurugu, Albert Doughan, Abdul-Rahman Adamu Bukari, Mahtaab Hayat, Emmanuel James San, Hannah Nyarkoah Nyarko, John Mogaka, Mwangi Harrison Ndung'u, Farzana Rahman, Sayane Shome

**Affiliations:** 1 Department of Integrative Biomedical Science, IDM, Faculty of Health Sciences, University of Cape Town, Cape Town, University of Cape Town, South Africa; 2 Department of Biochemistry and Biotechnology, Kwame Nkrumah University of Science and Technology, Kumasi, Ashanti, Ghana; 3 Department of Microbiology, University of Manitoba, Manitoba, Canada; 4 Sydney Brenner Institute for Molecular Bioscience, Faculty of Health Sciences, University of the Witwatersrand, Johannesburg, South Africa; 5 Division of Human Genetics, National Health Laboratory Service and School of Pathology, Faculty of Health Sciences, University of the Witwatersrand, Johannesburg, South Africa; 6 Kwazulu-Natal Research and Innovation Sequencing Platform (KRISP), College of Health Sciences, University of KwaZulu-Natal, Durban, South Africa; 7 Discipline of Public Health, University of Kwazulu-Natal, Durban, South Africa; 8 Biochemistry Department, Center for Biotechnology & Bioinformatics, University of Nairobi, HEP Bioinformatics Consultants LTD, Nairobi, Kenya; 9 School of Natural and Applied Science, Canterbury Christ Church University, Canterbury, Kent, UK; 10 Faculty of Computing, Engineering and Science, University of South Wales,, Wales, UK; 11 Bioinformatics and Computational Biology Program, Iowa State University, Ames, Iowa, USA

**Keywords:** Bioinformatics, Africa, Student Council Symposium, ISCB, ASBCB, Ghana

## Abstract

The article elaborates on the program highlights of the 3rd African Student Council Symposium 2019. The one-day symposium was held in Kwame Nkrumah University of Science and Technology (KNUST), Ghana, on 11 November 2019 during the 6th joint international bioinformatics conference of the ISCB and ASBCB. It consisted of three sessions that included keynote talks by Prof Christine Orengo and Dr. Amel Ghouila, and seven selected student speaker talks from different areas of bioinformatics. The students benefited from networking and learning about ongoing research work by their peers hailing from different countries of the African region. The symposium proved to be pivotal to strengthen connections in the African bioinformatics student community.

## Introduction

Major international genomics projects and advancement in genome sequencing technologies have brought in significant improvement in biological research and clinical medicine ^1^. It paved the era of enormous biological data generation, which in turn has facilitated scientific research in various domains such as pharmaceuticals, microbial forensics, agriculture, and personalized healthcare. For better inference from the data, the field now requires more researchers and specialists in the field of bioinformatics and computational biology, who can build and implement computational methods for faster and efficient data analysis. Through the efforts of organizations such as the International Society for Computational Biology (ISCB), African Society for Bioinformatics and Computational Biology (ASBCB) and the H3ABioNet consortium, capacity building amongst computational biologists has greatly improved in the African region.

The Student Council Symposium (SCS) is a one-day event with a prime focus on youth researchers to present their work at the event. Various student council symposia have been organized over the years across the world periodically ^2^. SCS Africa is organized in line with the ISCB Africa conference once every two years ^3, 4^. The third edition of SCS Africa was hosted on 11 November 2019 in collaboration with ISCB Student Council and the local SCS organizing team.

The symposium venue was at the Kwame Nkrumah University of Science and Technology (KNUST), Kumasi, Ghana. KNUST is the second largest university in Ghana, with a student population of about 57,000, located within the capital city of the Ashanti region. The conference venue was the Amonoo-Neizer conference center, well-known for hosting various major conferences.

SCS Africa aimed to create a conducive atmosphere for networking as well as provide participants a chance to interact with bioinformatics experts. On that premise, the symposium provided an excellent platform for young African researchers and students to present their work to an enthusiastic audience already pursuing (or considering) bioinformatics research. The participant pool ranged from undergraduate students to post-doctoral researchers working in different domains of bioinformatics research.

## Event proceedings

This year, similar to the format of previous symposia ^3, 4^, SCS Africa began with a welcome address from the conference organizers, followed by keynote addresses, an icebreaker, and six 10-minutes student presentations. The event ended by appreciating participants, sponsors, and organizers.

We observed attendance from researchers with a wide range of research backgrounds comprising 30 students from 8 different countries across the continent and one attendee each from Australia and the United States of America. A call for abstracts was made months before the meeting and reviewed by external volunteers. Abstract acceptance was sent out based on reviews and availability of travel fellowships.

The welcome address was delivered by Dr. Samson Pandam Salifu, who is the Principal Investigator of the H3ABioNet-KNUST node and Co-chair of the ISCB Africa ASBCB conference 2019. He welcomed all participants and discussed the relevance of bioinformatics research for development in Africa. Further, his talk highlighted the recent emergence of bioinformatics graduate programs in various institutions across Africa, such as in Ghana, Kenya, Nigeria, Egypt, and Uganda. He concluded the address asserting bioinformatics would grow as we explore more about the biological origins of life.

The first keynote session followed, which was by Professor Christine Orengo, a computational biologist whose core research has been the development of robust algorithms to capture relationships between protein structures, sequences, and functions. She has built one of the most comprehensive protein classification databases, CATH ^5^, used worldwide by tens of thousands of biologists, and central to many pioneering structural and evolutionary studies. Professor Orengo is also the President-Elect of the International Society of Computational Biology (ISCB) and the founder of the European ELIXIR 3D-BioInfo Community in Structural Bioinformatics. The title of her talk was "Looking for LUCA – and other evolutionary tales." Professor Orengo could not join the conference on Monday due to unforeseen circumstances and, hence, sent a pre-recorded video.

Professor Orengo’s talk consisted of a discussion of her current research as well as providing students with suggestions and career tips to excel in life. Her detailed presentation was segregated on four sub-topics: finding one’s focus, one’s courage, one’s friends, and catching the wave. She explained that as young scientists, it was essential to find the niche that fits our passions and skills and what drives us in our lives.

Professor Orengo started her life with chemistry and physics, studying small molecules. She moved to medical physics but later realized that she missed studying the small molecules. Hence, she started working again in biochemistry, particularly enzymes. She was also fascinated by the structures of proteins that regulate the enzyme functions.

In her address, she emphasized the importance of finding friends during one’s career with whom we enjoy working and collaborating. She gave an example from her personal life about how she found a collaborator working on protein structure algorithms that led to the CATH database development. At that point, she had caught the wave of sudden interest in genomic data interpretation. They obtained all the data available worldwide to draw inferences using evolutionary relationships and built the well-acclaimed CATH database. She said at a point she realized the spike in interest in protein sequences and integrated that into the CATH database, expanding structure superfamilies with sequence data. Using the Last Universal Common Ancestor (LUCA) approach, they found several protein families across species, which in turn formed the superfamily. Then a new wave leads to interest in the large-scale generation of gene expression data. It facilitated their ability to find the genes that were correlated either functionally or via expression levels. It was further used to build signaling or metabolic pathways by employing the Homology Inferred Protein-Protein Interactions algorithm, in collaboration with a research group in Germany working on protein-protein interaction in chromosomal condensation. Professor Orengo intimated about the current wave of data in personalized genomics involving massive sequencing initiatives in human gene variations. She has therefore found new collaborations in FunPDBe and Genomics England, GeCIP Functional Effect Domain investigating residue-site mutations in human genes to understand their disease-associations.

In her take-home reflections, Professor Orengo reiterated the need to find one’s focus, courage, taking risks, and trying new challenges, finding friends, collaborators, competitors in our life journeys. She encouraged participants to take risks and go for that grant or fellowship, set up teams, talk at that big meeting, and question accepted theories. In her closing words, she motivated the audience to explore the upcoming new technologies for data generation and to stay true and be generous. All questions apropos her presentation were addressed on Wednesday when she arrived.

The second keynote speaker was Dr. Amel Ghouila, a bioinformatician working for H3ABioNet to help build capacity in bioinformatics throughout Africa. As part of H3ABioNet activities, Dr Ghouila leads the Big Data Analytics and Machine Learning (ML) project focusing on exploring the use of ML tools to improve health outcomes. She also coordinates the sustainability and outreach activities within H3ABioNet, aiming at developing strategic public outreach plans, including audiences such as governments, patients, and youth. She is the general secretary for the ASBCB and is also the founder of Technovation Tunisia, which is a non-profit entity dedicated to teaching young girls coding and entrepreneurship skills while addressing the current challenges in their communities. The title of her talk was "Leveraging open science and collaborative research for better health outcomes."

Dr. Ghouila shared her experiences about open science research and collaborative science in Africa. Her interest in bioinformatics grew when she gave a talk in 2007 at an ASBCB organized conference, where she discussed some of her research findings without much background knowledge about the terminologies in the field of bioinformatics.

The conference was a good turning point for her career. She had the opportunity to network with her Ph.D. supervisor and her current colleagues. She, therefore, urged participants to talk and interact with the next person as that can lead to future collaborations and research.

After her Ph.D., Dr Ghouila had various questions in mind, including the dilemma about the career to pursue ahead. As she started attending job interviews for academic positions, she realized the path to success in academia is rockier than the one she had imagined. That made her question her actual intent for her future career endeavors. The introspection made her realize her interest in impacting public health in Africa, where she observed a major disconnect between efforts done by academia to actual requirements in public health. She then started thinking of how to challenge the research ecosystem and how researchers can work together to improve health.

At the same time, she heard of the Mozilla Open Science group, which aims to connect researchers all over the world, primarily focused on public health to share skills and conduct meetings with participants from different countries. She started her open science research group in Tunisia, consisting of several researchers from different backgrounds working on a joint project.

In 2016, Dr Ghouila received an invite to be part of the first Mozilla cohort in Berlin that year, which was another turning point in her career. She spent more time advocating open science and open data that shifted her thinking about classic academic researchers’ thinking. Her project took advantage of the evolution of technology to enhance their collaboration as they started using phones, computers, watches to communicate. Coincidentally, the cost of sequencing went down over the years enabling more generation of data which needs a lot of skills to analyze. She also noted one needs to keep on updating their skill set as the time passes. The scientific research ecosystem has become more complex with time, as technology advances and data collection procedures are becoming more diverse. Considering all of this, researchers need to apply comprehensive approaches that help in disease prevention and benefit the communities. She also highlighted that while achieving these objectives, one also needs to consider the ethical considerations and critique the research methods used. She further explained the importance of the reproducibility of research results by methods used and the concept of open science research.

The striking point in Dr Ghouila’s presentation was the analogy of blind people and the big elephant. In her story, blind people were asked to touch the elephant from different positions and to describe what they touched. Some touched the ears, legs, trunk, and other parts. The moral of the analogy is we have to remember that what we observe is not nature itself, but nature exposed by our methods of questioning. She quoted Werner Heisenberg's quote, “We all observe things from different angles, and we do not have the totality of the truth because we all come with a piece of the truth from the angle of our understanding.”

She concluded the story suggesting that if we all come together to collaborate, we might come out with better solutions and answers to different questions we ask, which highlights the importance of open science and collaborations. She shared tools and open science projects such as the African Open Science Platform, which promotes scientists to collaborate more and contribute, and further talked about open access, preprint, research communication, community engagement, and her NGO for young girls in science.

Dr Ghouila concluded her talk by encouraging the participants to work beyond the research pipeline, think about what they enjoy doing while connecting the dots between their research projects, and how they can contribute as scientists for the benefit of humanity.

## Student presentations

During the student presentation session, six graduate students and one lecturer gave an oral talk for 10 mins on their research works. Below is a brief description of the talks.

San James, from the Kwazulu-Natal Research, Innovation and Sequencing Platform (KRISP) at the University of Kwazulu Natal, gave the first presentation on a computational method for the identification of Dengue, Zika and Chikungunya virus species and genotypes ^6^. The method employs phylogenetic analysis to classify the viruses accurately. It has been implemented in the Genome Detective ^7^ framework, which provides an intuitive, fast, and automated web-based platform. He further intimated that the tool is useful to quickly and easily understand the outbreaks of these arboviruses and provide a robust platform for developing other genotyping tools.

James Abugri, from the University for Development Studies, Ghana, presented his work on "Population genomics of *Plasmodium falciparum* Clinical Isolates in Ghana" He discussed local population differentiation within two towns in Ghana with differing transmission intensities. The study also investigated polygenomic infections and signatures of selections. The selection scans leveraged haplotype-based metrics to examine signatures of antimalarial drug resistance ^8^. The study also revealed signatures around the chloroquine-resistant gene and the antifolate drug-resistant genes. The work was supported by funding from a Leverhulme-Royal Society Africa Award (Grant AA110050), an ERC Advanced award (Grant AdG-2011-294428), the UK Medical Research Council (G1100123 and G0600718), and the Wellcome Trust (090770/Z/09/Z).

Gilbert Kibet-Rono, an MSc in Bioinformatics student at the International Centre of Insect Physiology and Ecology, Kenya, presented his master's thesis work on "Phylogenetic and phylogeographic meta-analysis of cytochrome C oxidase I barcode sequences of African arthropods submitted into the Barcode of Life Database." He gave a detailed description of a bioinformatics workflow developed to address the limitations faced in African arthropods phylogeographic research. The workflow relies on BOLD data, Ribosomal-Database-project Classifier to identify sequences, and an assembly of bioinformatics tools and scripts in R, Bash, Python, among other languages. This workflow will support the research and understanding of evolutionary and population dynamics of insects leading to better conservation efforts, pests, and vector management.

David Twesigomwe from the University of the Witwatersrand, Johannesburg, South Africa, presented his work funded by GlaxoSmithKline titled "Characterisation of CYP2D6 Pharmacogenomic Variation in African Populations: an Integrative Bioinformatics Approach". He first gave a comparison of three algorithms (i.e., Astrolabe, Aldy, and Stargazer) for calling star alleles in CYP2D6 and other pharmacogenes. He showed that all three algorithms have high analytical sensitivity. However, Astrolabe has a lower recall for CYP2D6 CNVs, and Stargazer has a lower recall for rare alleles. At the same time, Aldy and Astrolabe are liable to ambiguous calls when sub-variants are not well-defined. David also presented preliminary findings from genotyping CYP2D6 using H3Africa's high coverage of African genomes and the three algorithms.

Haward Ketoyo from KNUST, Ghana, presented his work on "Development of an Artificial Neural Network (ANN) classifier for classification of Breast Cancer Metastasized Sites." He described an ANN model, which could classify primary sites from cancer sites primarily invaded by breast cancer. Four classifiers were developed based on different features in the dataset. He concluded that the best performing classifiers had genes and mutation descriptions as the only features in the data set for training with an accuracy of 77%.

Wisdom A. Akurugu, a Ph.D. candidate from the University of Cape Town, South Africa, presented his research work entitled "The Role of rs41423247 in Protection Against Hypothalamic-Pituitary-Adrenal (HPA) Axis Suppression (HPAS) in Asthmatic Children on Corticosteroids". He indicated that inhaled corticosteroids (ICS) are one of the most effective medications for asthma treatment but can lead to adrenal suppression if used for long periods and at high dosage. Recent studies had revealed such suppression among children on ICS medication. He also indicated that single nucleotide polymorphisms (SNPs) in genes involved with the HPA axis affect various levels of cortisol production. Wisdom aimed to determine if three of such SNPs were associated with HPAS in children on corticosteroid medications using Body Mass Index (BMI), Cortisol, adrenocorticotropic hormone (ACTH) as indicators. Using both statistical and bioinformatics resources, Wisdom showed that one of his focal SNPs, rs41423247 was protective against HPAS and associated with BMI. The protective effect was, however, independent of BMI. The homozygous variant genotype (CC) was associated with higher post-metyrapone ACTH. The genetic effect was inherited dominantly and that the SNP has a regulatory impact on its gene, the nuclear receptor subfamily 3 group C member 1 (NR3C1). Wisdom's research work received financial support from the South African National Research Foundation (NRF) and the South African Thoracic Society.

The final presenter was Isabel Mensah, a Ph.D. student from KNUST, Ghana, who presented on "Wavelets based feature extraction with principal component analysis for predicting autism in neonates using the Bayesian classifier." She utilized mathematical methods for denoising data and selecting some relevant genes that contribute to the possibility of Autism Spectrum Disorders (ASDs). Isabel also discussed some machine learning techniques used to predict the presence of the disease in an individual as well as their possible severity. She stated that approximately 200 differentially expressed genes were identified and used for predicting ASD status of children with a classification accuracy of 95.91%. She concluded that, by optimizing and implementing these models in clinical settings, the health burden of ASDs might be significantly reduced. The work was funded by Petroleum Geo-Services through the National Institute for Mathematical Sciences, Ghana.

## Awards

The presentations were judged by Professor Alia Benkhala, the president of ASBCB, Professor Faisal M. Fadlelmola, an H3ABioNet Principal Investigator, and Albert Doughan, a member of the organizing committee, based on the following criteria: body language and eye contact, appropriate vocabulary and grammar, clear aim and objectives, well-described methods, key results with interpretation, visual aids, overall impression and ability to answer questions.

Wisdom A. Akurugu emerged as the overall winner. The first and second runners up were David Twesigomwe (University of Witwatersrand) and Isabel Mensah (KNUST), respectively.

The best presenters were awarded prizes in cash and books by the president of ASBCB, Professor Alia Benkhala. Stoles made with the colourful Ghanaian Kente material were also presented to Dr. Amel Ghouila and Professor Christine Orengo as appreciation for their keynote talks.

## Post-symposium survey

Presenters and audience of the symposium were contacted via e-mail post-symposium to obtain feedback of their experience of the symposium. The feedback intended to assess the impact the symposium had on the participants and to provide recommendations for future symposia. Overall, we received eight responses, out of which seven had never attended any ISCB Africa Student Council Symposium before. Respondents were highly satisfied with both the keynote addresses and the presentations. A respondent suggested for more vigorous and earlier promotions for ISCB student council symposia to attract more participants. All respondents were eager to recommend our events to friends or colleagues.

## Conclusion and recommendations

The co-chair of the organizing committee, Mr. Wisdom Akurugu, thanked Professor Orengo and Dr Ghouila for their time and inspirational talks. Further, he appreciated the time and efforts of presenters, participants, organizing committee that made the symposium a success.

The SCS Africa symposia is an excellent base to showcase the growing bioinformatics capacity in Africa and motivates undergraduate students to pursue the bioinformatics career path. The networking and collaboration opportunities established among students from a broad research background from across Africa can be useful in addressing future challenges that require bioinformatics expertise. It is, therefore, important that future SCSs continue to be dynamic by introducing activities that will continue to enhance student's interaction with peers and researchers from industry and academia. This number of student presenters could have been increased with an earlier set timeline for abstract call and vigorous promotions. However, funding constraints are the major bottleneck. Indeed, there is a need for a sustainable approach for funding future symposia. In addition, African institutions need to establish more travel support and invest more in such ventures for their students seeking to present their works at a national and international level.

## Data availability

No data is associated with this article.
